# Quantitative iTRAQ LC-MS/MS reveals muscular proteome profiles of deep pressure ulcers

**DOI:** 10.1042/BSR20200563

**Published:** 2020-06-15

**Authors:** Zan Liu, Xu Cui, Yanke Hu, Pihong Zhang

**Affiliations:** 1 Department of Burns and Reconstructive Surgery, Xiangya Hospital, Central South University, Changsha, Hunan, P.R. China; 2 Department of Pediatric Surgery, Hunan Children’s Hospital, Changsha 410004, Changsha, Hunan, P.R. China; 3 Institute of Burn Research, Xiangya Hospital, Central South University, Changsha, 410008.Hunan Province, P.R. China

**Keywords:** lysosome, muscle, pressure ulcers, proteomic

## Abstract

Pressure ulcers (PUs) are a common clinical issue lacking effective treatment and validated pharmacological therapy in hospital settings. Ischemia–reperfusion injury of deep tissue, especially muscle, plays a vital role in the formation and development of the overwhelming majority of PUs. However, muscular protein expression study in PUs has not been reported. Herein, we aimed to investigate the muscular proteins profiles in PUs and to explore the pathological mechanism of PUs. The iTRAQ LC-MS/MS was conducted to detect the protein profiles in clinical muscle samples of PUs. The GO and KEGG pathways analyses were performed for annotation of differentially expressed proteins. Protein–protein interaction (PPI) network was constructed by STRING online database, and hub proteins were validated by the immunoblotting. Based on proteomics results, we found a number of proteins that were differentially expressed in PU muscle samples compared with the normal and identified unique proteins expression patterns between these two groups, suggesting that they might involve in pathological process of the disease. Importantly, cathepsin B and D, as well as other autophagy–lysosome and apoptosis associated proteins were identified. Further experiments characterize the expression of these proteins and their regulation in the process of apoptosis and autophagy. These findings may provide novel insights into the mechanisms of lysosome-associated pathways involved in the initiation of PUs. This is the first study linking proteomics to PUs muscle tissues, which indicated cathepsin B and D might be key drug target for PUs.

## Introduction

Pressure ulcers (PUs), also known as pressure sores or bedsores, are quite common in acutely and chronically ill patients and usually occur in the soft tissues directly covering the bony prominence leading tissue necrosis as a result of pressure or pressure in combination with shear and/or friction [1]. As a complication of immobility among the intensive care and spinal cord injury patients or elderly, PUs have serious consequences on patient morbidity and mortality, as well as the cost of care [2]. To date, attempts to deal with PUs, such as bone and soft tissue excision, and coverage with a flap, fail to produce a significant improvement of the health problem [3]. Additionally, the benefit of using systemic or topical antibiotics in the management of PUs is still unclear [4]. More research is needed to assess how to best support the treatment of PUs.

The formation of PUs is considered to be multifactorial. Besides the magnitude and duration of the pressure, risk factors such as shear force on the skin, nervous function, local blood circulation, age, nutritional condition and accompanied diseases also contribute to the development of PUs [5–7]. However, basic studies for elucidating the etiology of the clinical condition and the mechanisms of the ulcer formations are limited. Recently, the occurrence of cycles of ischemia–reperfusion (I/R) has been used wildly as a physiological relevant inducer to study PUs [8], and increasing evidences suggest that I/R injury of deep tissue, especially muscle, plays a crucial role in the formation and development of the overwhelming majority of PUs [9,10]. Tissues deprived of blood supply during an ischemic attack reduce their metabolism in an effort to maintain tissue function. Recovery of blood flow to hypoxic and nutritionally deficient tissues (i.e. reperfusion events) can trigger a series of events due to a sudden increase in oxygen free radicals. Cytotoxic effects of excess free radicals lead to severe disorders of inflammation and recruitment of cells to the site of injury [11]. Previous animal model studies have shown that repeated I/R cycles of skin tissues result in escalated synthesis of reactive oxygen species (ROS), while ROS are not eliminated by local oxygen free radical scavengers, leading to increased inflammatory response and skin necrosis [12]. In wounded tissues, the dysregulated synthesis of ROS by inflammatory cells exerts deleterious effects on lipids, proteins and nucleic acids of cells involved in wound repair leading to tissue damage [13]. Meanwhile, leukocytes become activated and invoke an inflammatory cascade, causing cellular edema and tissue damage. Besides, the initial metabolic event during tissue ischemia is involved in disruption of energy metabolism and mitochondrial dysfunction, which activates apoptotic pathways. Previous studies have showed apoptotic-related proteins, such as Bax and HIF-1 (hypoxia inducible factor 1), play an important role in different processes of pressure ulceration (either early stage or healing stage) [14,15].

On grounds of continued high rates of occurrence and challenges in treatment of PUs, it is still urgent to develop more innovative and effective treatments targeting cellular and molecular mechanism underlying the pathogenesis of PU formation [16]. Recently, advances in mass spectrometry-based proteomics have enabled the measurement of multiple properties of thousands of proteins, including their abundance, subcellular localization, post-translational modifications, and interactions [17]. While traditional molecular biology methods detect a limited number of proteins based on signaling or metabolic pathways, proteomics has become a systematic approach to qualitative and quantitative localization of entire proteomes in large-scale research [18]. In the area of traumatology, proteomics for comparing protein expression profiles between normal and disease states has seldom been applied to obtain unique protein-expression profiles of PUs, let alone deep tissues. The present study aimed to use proteomic approaches to carry out fundamental biological studies to explore mechanism underlying on clinical samples.

## Methods

### Study subjects

Eight patients with deep PUs undergoing surgical treatment in our department were enrolled between January 2016 to December 2017, the clinical characteristics of PU patients are presented in Supplementary Table S1. PU muscle tissue samples were obtained from surgical specimens of patients, and normal muscle tissues were harvested from patients undergoing amputation or flaps operation. Tissue samples were rinsed twice with ice-cold PBS and transferred to liquid nitrogen immediately. The study was conducted in accordance with the Declaration of Helsinki and approved by the Ethics Committee of Xiangya Hospital, Central South University, and all patients gave informed consent to participate in the present study.

### H&E staining

H&E staining were performed in paraffin-embedded pressure ulcer muscles sections. The waxes were sectioned serially at 5-µm thickness. After deparaffinization and rehydration, standard H&E staining was carried out to visualize the pathological characteristics of the muscles.

### Protein preparation and iTRAQ-LC-MS/MS

The tissue samples were grinded by liquid nitrogen into cell powder and then disintegrated by lysis buffer (8 M urea, 1% Protease Inhibitor Cocktail). Followed by sonication three times on ice using a high intensity ultrasonic processor (Sonics, U.S.A.) and centrifugation at 12,000 ***g*** at 4°C for 10 min, the supernatant was collected and the protein concentration was determined with BCA kit (Thermo Scientific, Rockford, U.S.A.) according to the manufacturer’s instructions. Then, the protein solution was reduced with 5 mM dithiothreitol and alkylated with 11 mM iodoacetamide. After the urea concentration was diluted to less than 2M, trypsin was added for digestion. The digested peptides were subsequently labeled with iTRAQ (isobaric Tags for Relative and Absolute Quantification) reagents following the manufacturer’s instructions. Then, the iTRAQ-labeled sample mixtures were used to conduct liquid chromatography–tandem mass spectrometry (LC–MS/MS) experiments using an EASY-nLC 1000 UPLC system as follows: The peptides were subjected to NSI source followed by tandem mass spectrometry (MS/MS) in Q Exactive™ Plus (Thermo) coupled online to the UPLC. The electrospray voltage applied was 2.0 kV. The *m*/*z* scan range was 350–1800 for full scan, and intact peptides were detected in the Orbitrap at a resolution of 70,000. Peptides were then selected for MS/MS using NCE setting as 28 and the fragments were detected in the Orbitrap at a resolution of 17,500. A data-dependent procedure that alternated between one MS scan followed by 20 MS/MS scans with 15.0 s dynamic exclusion. Automatic gain control (AGC) was set at 5E4. Fixed first mass was set as 100 *m*/*z*. The resulting MS/MS data were processed using Maxquant search engine (v.1.5.2.8), the FDR for protein identification and PSM identification was set to 1%.

### Bioinformatics analysis

The biological function of differentially expressed proteins was identified by Gene Ontology (GO) and Kyoto Encyclopedia of Genes and Genomes (KEGG) pathway enrichment analyses. GO annotation proteome was derived from the UniProt-GOA database (http://www.ebi.ac.uk/GOA/). Proteins were classified by GO annotation into three categories: biological process, cellular compartment and molecular function. KEGG connects known information on molecular interaction, reaction and relation networks, such as pathways and information about genes and proteins generated by genome projects. Fisher’s exact test was employed, and the occurrence of false positives was corrected by Benjamini–Hochberg (B-H) multiple test correction method. An adjusted *P* value of < 0.05 was set as the cut-off criterion. To observe significantly enriched pathways, pathway mapper was used for coloring of differentially expressed proteins with different color. Thus, each search object is specified in one line together with color attributes.

### Western blot analysis and antibodies

The immunoblotting experiment was performed as previously described [19,20]. In brief, total protein was isolated from tissue samples using RIPA lysis buffer with protease inhibitor cocktail tablets (Roche, Switzerland), and quantified using a BCA Protein Assay Kit (Thermo Fisher Scientific, U.S.A.). The total protein samples were loaded and separated on TGX Stain-Free™ FastCast™ Acrylamide Kit (Bio-Rad, U.S.A.) and transferred to PVDF membranes (Merck Millipore, Germany). The membranes were blocked with 5% skim milk for 2 h and incubated with primary antibodies against cathepsin D (Abcam, U.S.A., 1:2000), Bax (ProteinTech, China, 1:200), cathepsin B and Bcl-2 (Cell Signaling Technology, U.S.A., 1:1000) overnight at 4°C, which was followed by incubation with the corresponding secondary antibodies for 2 h at room temperature. Signals were visualized by enhanced chemiluminescence (ECL) reagents (Abvansta, U.S.A.) and captured by a Chemi Doc^MP^ Imaging System (Bio-Rad, U.S.A.). Total protein was used for normalization. Immunoreactive bands were quantified using ImageJ.

### Statistical analysis

All data are presented as mean ± standard deviation (SD). Statistical analysis was performed using unpaired Student’s *t*-test and GraphPad Prism software was used to perform the statistical analyses (GraphPad Prism version 6.0, San Diego, CA, U.S.A.). The values of *P*<0.05 were considered statistically significant for all tests.

## Results

### Global profiling of proteins in human PU muscle

**Figure 1:**
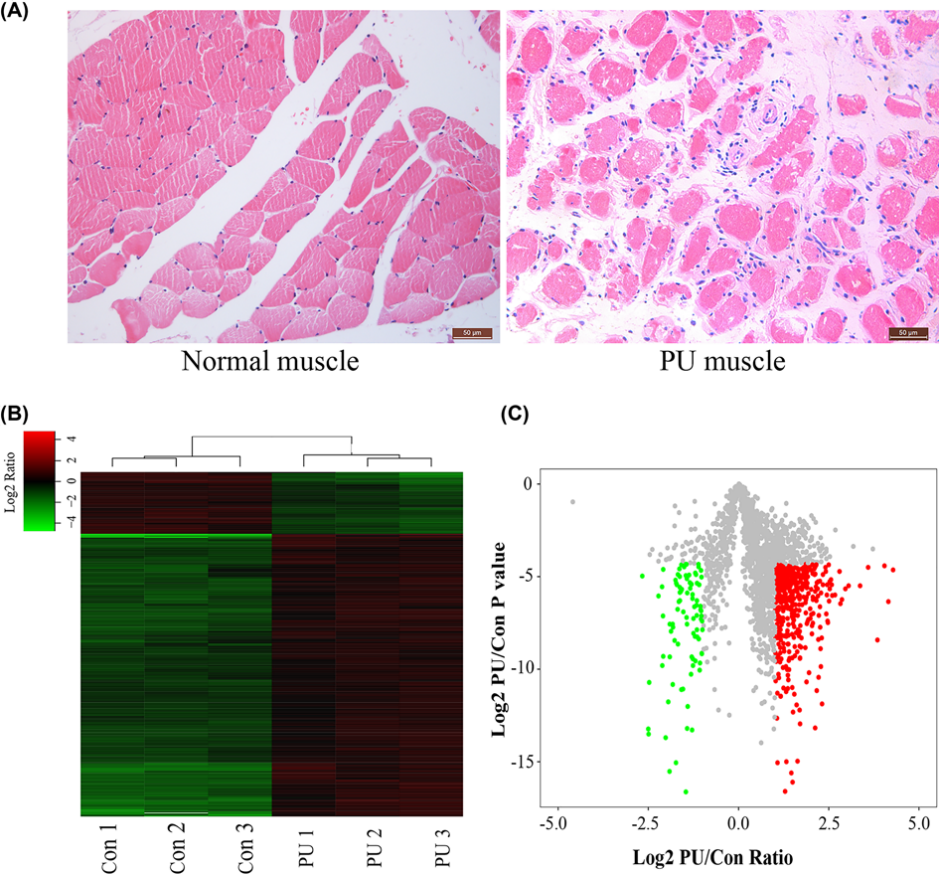
Morphology and proteome files of PU muscle tissues (**A**) HE staining of normal muscle and pressure ulcer muscle tissues (200×). (**B**) Hierarchical clustering of differentially expressed proteins. (**C**) Volcano plots of all proteins identified in LC-MS/MS analysis, red dots in the plots represent the up-regulated proteins with statistical significance, and green dots in the plots represent the down-regulated (Fold change ≥2.0, *P*<0.05).

To gain better insight into the full spectrum of pathological alterations initiated by PUs at the molecular level, we performed a proteomics analysis on PU muscle tissues and normal muscle samples. Pathological features such as atrophied or fractured myofibers with a rounded shape, increased endomysium distance between the fibers of PU muscles were demonstrated by H&E staining as shown in Figure 1A. In total, 2558 proteins were identified in the present study, 520 of which were identified with differential expression between samples from patients with deep PU and normal muscle tissues (fold change ≥2, *P*≤0.05; the list of differentially expressed proteins after mass spectrometry was shown in Supplementary Table S2). To investigate the expression patterns of proteins, hierarchical clustering was performed to analyze proteins expression in clinical samples of PU patients. The data showed a distinguishable protein expression profiling pattern between PU muscles and control groups (Figure 1B). To identify differentially expressed proteins, Volcano Plot analysis was conducted to visualize the difference between PU muscles and control groups (Figure 1C).

### *ln silico* analysis of differentially expressed proteins

**Figure 2:**
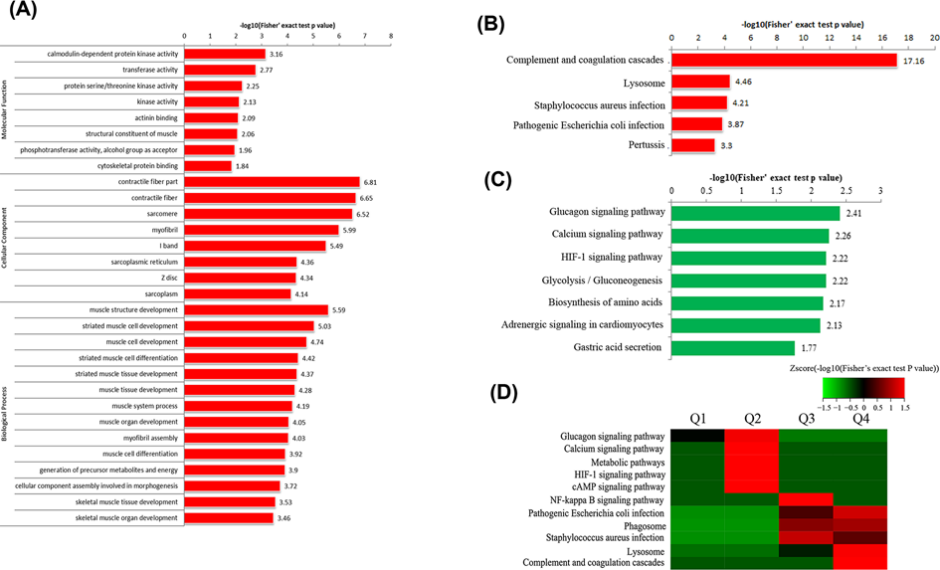
Bioinformatics analysis of the differentially expressed proteins in PU muscles (**A**) Enrichment of Gene Ontology analysis. (**B**) KEGG pathways for up-regulated proteins in PU muscle. (**C**) KEGG pathways for down-regulated proteins in PU muscle. (**D**) Comprehensive KEGG pathway analysis of the differentially expressed proteins based on the difference multiple, Q1 (0< Ratio ≤ 1/3), Q2 (1/3 < Ratio ≤ 0.5), Q3 (2 < Ratio ≤ 3) and Q4 (Ratio > 3).

Enrichment of Gene Ontology (GO) analysis depicting differentially expressed proteins based on three categories are shown in Figure 2A. We found a number of differentially expressed proteins and identified unique proteins expression patterns between PU muscles and normal samples based on proteomics results. The muscle structure development and muscle cell development were highly enriched in biological process category, suggesting the abnormal muscle structure and dysfunction of PU muscles, which was verified by H&E staining. Subsequent KEGG analysis of the up-regulated proteins indicated that the complement and coagulation cascades and the lysosome were highly enriched (Figure 2B). While the glucagon signaling pathway and the calcium signaling pathway were highly enriched in down-regulated proteins in PU muscles (Figure 2C). For further decipher the related information of protein function of different differential multiples, we further divided the differential expressed proteins into four parts: Q1 (0< Ratio ≤ 1/3), Q2 (1/3 < Ratio ≤ 0.5), Q3 (2 < Ratio ≤ 3) and Q4 (Ratio > 3). As shown in Figure 2D, deeper analysis of the most differentially expressed proteins in PU muscles still highlighted the complement and coagulation cascades and the lysosome as the most changed pathways on the basis of the number of changed proteins and statistical significance. In addition, we found signaling pathways associated with inflammatory signal transduction and immune response were changed at protein level, including the staphylococcus aureus infection, HIF-1α and NF-kappa B signaling pathway (Figure 2D). This is consistent with previous reports that bacterial infection and tissues inflammation play an important role in the pathogenesis of PUs, and also provided more comprehensive details of pathway dynamics at protein level.

### The lysosome-associated pathways in PU muscles

Lysosomes are capable of fusing with other organelles and digesting large structures or cellular debris through cooperation with phagosomes, endosomes and autophagosome [21,22]. The unbiased KEGG pathway enrichment analysis based on differentially expressed proteins uncovered specific, robust and early reprogramming of lysosome-associated pathways in the PU muscles. The metabolic derangement of lysosome can induce apoptosis and autophagic cell death by regulating relevant signaling transduction pathways. Notably, PU muscles elicited a highly unique gene expression profile, in which proteins involved in phagocytosis, endocytosis, apoptosis and autophagy pathways were identified as dramatically changed in comparison to the normal group (Supplementary Figure S1A–D). These lysosome-linked biological processes have been seen as an adaptive response to stress, which promotes survival, whereas in other cases it appears to promote cell death and morbidity.

### The up-regulation of Cathepsin B/D and their PPI analysis

**Figure 3:**
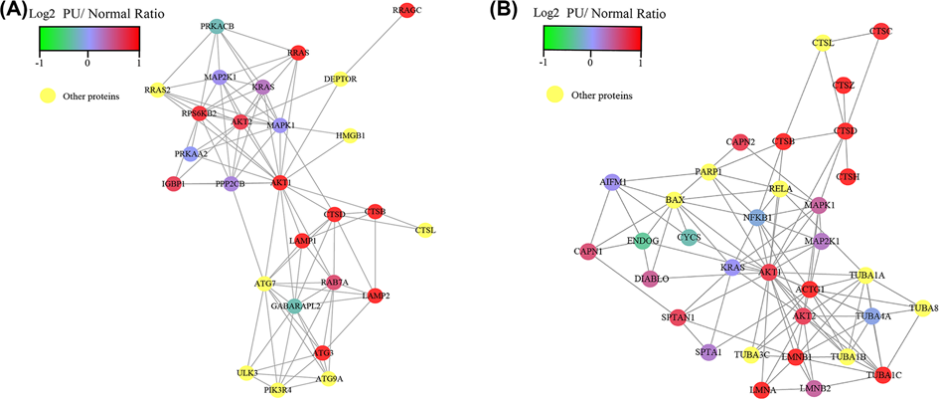
The protein–protein interaction (PPI) of autophagic or apoptotic proteins (**A**) PPI of autophagy-associated proteins obtained from the STRING database with a confidence score of ≥ 0.7 containing 27 nodes and 95 edges. (**B**) PPI of apoptosis-associated proteins obtained from the STRING database with a confidence score of ≥ 0.7 containing 33 nodes and 103 edges.

Among those changed proteins involved in the lysosome-associated pathways, cathepsin B (CTSB) and cathepsin D (CTSD), belonging to a family of lysosomal cysteine proteases that play an important role in intracellular proteolysis, were upregulated proteins in PU muscle tissues (Supplementary Table S2). Proteins rarely act alone as their functions tend to be regulated; therefore, we used protein–protein interactions (PPIs) to identify the interacted proteins responsible for cathepsin B/D. Based on the PPIs analysis, several proteins interacted with CTSB and CTSD were also up-regulated in PU muscles, such as RRAS, Rab7, AKT1, AKT2, LAMP1, LAMP2, ATG3 and ATG7, which were involved in autophagy–lysosome signaling pathway (Figure 3A). In addition, proteins participating in apoptosis signaling pathway were shown in PPI network, which included BAX, CTSZ, ACTG1, LMNB1, AKT1 and AKT2 (Figure 3B). To obtain evidence of bioinformatics analysis supporting the role of cathepsin B/D in lysosome-associated pathways, we then searched pathways involved in cathepsin B/D by KEGG genes database and found that proteins encoded by *CTSB/CTSD* mainly involving in the autophagy, lysosome and apoptosis signaling pathways (Supplementary Figure S2A–C). Taken together, autophagy–lysosome dysfunction and apoptosis were considered as the important pathogenic events in PU development.

### Cathepsin B/D may participate in autophagy and apoptosis of PU muscles

**Figure 4:**
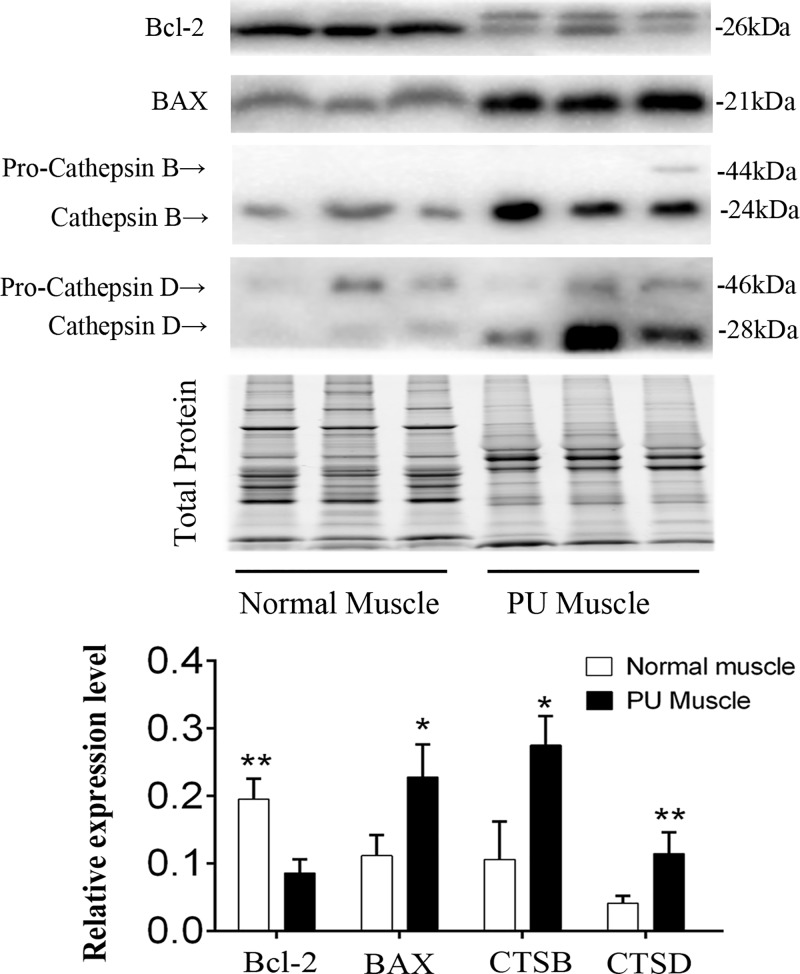
Immunoblotting of cathepsin B/D related proteins Representative immunoblot images of CTSB, CTSD, BAX and Bcl-2 expression in PU muscle by immunoblotting. TGX Stain-Free™ FastCast™ Acrylamide Gel was performed to assess the total protein expression, which was used for normalization (*n*=5; Means ± SD; *P<0.05, **P<0.01).

In accordance with PPI analysis, the level of cell death (apoptosis and autophagy) was elevated in PU muscles, as evidenced in our previous work [19]. To explore the causative role for the changes of protein expression associated with autophagy and apoptosis processes, we validated cathepsin B/D protein expression in muscle tissues from individuals with PU and the normal (Figure 4). In previous studies, up-regulation of CTSB is found in premalignant lesions and various pathological conditions including autophagy and apoptosis [23,24]. CTSB can enhance the activity of other protease, including matrix metalloproteinase, urokinase and CTSD [25], and thus has an essential position in the process of proteolysis. To gain a better understanding of how cathepsin B/D promotes PU, we next analyzed the expression of Bcl-2 and BAX, production of cathepsin B/D, in PU muscles and found that the Bcl-2 family protein levels were remarkably dysregulated relative to the normal (Figure 4), indicating that cathepsin B/D might regulate apoptosis by linking with pro-apoptotic or anti-apoptotic proteins. Taken together, autophagy–lysosome dysfunction and apoptosis were considered as the important pathogenic events in PUs and cathepsin B/D might play an important role in these biological processes.

## Discussion

With the aim of identifying critical pathological mechanism as new therapeutic targets for PUs, we demonstrate that overexpression of cathepsin B/D in PU muscles is closely related with dysfunction of autophagy-lysosome and apoptosis. The potential signaling mediated by this newly identified regulator in apoptosis was Bcl-2 family proteins in PU process. Participation of cathepsin B/D in lysosome-associated pathway could promote pathological process of PU. Thus, our study indicates that cathepsin B/D is a key drug target and its inhibitors may be potential therapeutics for PUs.

The PUs, a typical chronic non-healing wounds, are a serious health problem that develop mainly in elderly and immobilized patients, placing heavy burden to patients’ families and society. Recently, studies highlighted establishment of PU animal model to identify a signaling pathway that could be helpful to find a therapeutic target to prevent the deterioration of PUs in immobilized patients [26]. However, the mechanism by which these drugs may affect muscles impairment and wound healing of PUs is still undetermined [27]. Thus, we considered that proteomics could be a good alternative to identify the molecular mechanism associated with pathogenesis of PUs or explore the potential therapeutic agents in treating PUs.

The pathogenesis of PUs is mainly attributed by cycles of ischemia followed by reperfusion injury. Many studies have employed mouse model of I/R induced lesions to find a critical factor or a signaling pathway involved in PUs formation. For instance, monocyte chemoattractant protein-1 (MCP-1) participates in regulation of macrophage infiltration and subsequent skin inflammation during skin I/R injury [28]. In I/R injury murine model, interleukin-17 (IL-17) expression is found to be associated with severity of PUs [29]. Up to now, many innovative agents have been proposed. For example, selective inhibition of COX-2 by celecoxib has been shown to promote wound healing of PUs by reducing iNOS expression [30]. Olive oil can accelerate the resolution of PUs lesion in mice through inducing reduction of oxidative damage and inflammation [31]. Instead of animal models, we first carried out a proteome analysis in human PU and healthy muscles tissues. Many differentially expressed proteins and related pathological features identified previously were confirmed in our study, such as those associated with inflammation, oxidative stress and apoptosis. More importantly, the construction of protein profiles and PPI networks allowed us to identify the new proteins and biological events contributing to the pathogenesis of PUs.

Previous studies [19,32,33] have tested apoptotic factors of PUs and showed that apoptosis was involved in different processes of pressure ulceration. A recent study demonstrated that HIF-1α initiated mitochondria-mediated apoptotic pathways through the regulation of Bcl-2 family [15]. Bcl-2 antagonizes apoptosis, whereas Bax mediates apoptotic cell death. In our previous work, we found that both autophagy and apoptosis were elevated in PU muscles [19]. Interestingly, lysosome-related proteins cathepsin B/D were increased significantly in PU muscles, both of which were included in PPI networks associated with apoptosis and autophagy. It suggested that CTSB and CTSD should play an important role in development of PU. Some studies showed that CTSB contributed to traumatic brain injury-induced cell death through a mitochondria-mediated apoptotic pathway [24] and could be a key drug target [34]. However, no study has explored the role of cathepsin B in the formation of PU. Dramatically, consistent with CTSB and CTSD, protein levels of AKT were also up-regulated, as indicated in proteomics result. On the one hand, in apoptosis signaling pathway, AKT could phosphorylate a pro-apoptotic protein of the Bcl-2 family, Bad or Bax, which makes Bax dissociate from the Bcl-2/Bcl-X complex and lose the pro-apoptotic function [35]. On the other hand, both autophagy-related proteins ATG3, ATG7 and lysosome-related proteins LAMP1 and LAMP2 were significantly differential expression between the two groups. Previous paper has showed that AKT regulate lysosomal biogenesis and autophagy by direct phosphorylation of transcription factor EB [36]. Thus, the fact that AKT are considered as an indirect regulator could be explained.

Of note, complement and coagulation cascades, along with inflammatory and oxidative stress signaling pathways, were also significantly enriched in PU muscles by KEGG pathways analysis. It is not difficult to see that the inflammation and oxidative stress play an important role in PU pathology. Previous studies have indicated that the coagulation cascade was activated in skeletal muscle I/R injury, evidenced by that fibrin and thrombin deposition were significantly elevated [37]. Growing evidence has shown that the activity of the complement system is remarkably complicated and is implicated in inflammatory, neurodegenerative, age-related, and ischemic diseases [38]. Virtually, the complement system is readily activated during tissue injury to cause inflammation [39]. Studies have shown that inhibitor of complement system exerted muscular protective role on ischemia–reperfusion injury. Studies have shown that inhibitor of complement system exerted protective role on muscular ischemia reperfusion injury [37,40]. Nevertheless, the precise mechanism of complement and coagulation cascades in PU formation and development warrant further study.

## Conclusions

In summary, PU may finally result in biological dysfunction of tissues and cells, such as cell death, autophagy and inflammation. Therefore, our study on the role of cathepsin B/D in PU-induced cell apoptosis and autophagy (or lysosomal cell death) may offer a new strategy for the development of therapeutic interventions for PUs. However, some limitations in the present study have to be mentioned for future research. For examples, the molecular mechanisms and signaling pathways of apoptosis and autophagy regulated by cathepsin B/D are needed to further elucidate.

## Supplementary Material

Supplementary Figures S1-S2 and Tables S1-S2

## Data Availability

The data used to support the findings of this study are included within the article.

## Abbreviations

CTSB: cathepsin B
CTSD: cathepsin D
HIF-1: hypoxia inducible factor 1
I/R: ischemia–reperfusion
iTRAQ: isobaric Tags for Relative and Absolute Quantification
LC–MS/MS: liquid chromatography–tandem mass spectrometry
PPI: protein–protein interaction
PUs: pressure ulcers
ROS: reactive oxygen species
