# Supplementary material for: Quantitative iTRAQ LC-MS/MS reveals muscular proteome profiles of deep pressure ulcers
Source: Biosci Rep. 2020 Jun 15;40(6):BSR20200563. doi: 10.1042/BSR20200563 (PMC7295623; doi:10.1042/BSR20200563)
Supplement: Supplementary Figures S1-S2 and Tables S1-S2 [file BSR-2020-0563_supp.pdf]

Supplementary Figure 1

A

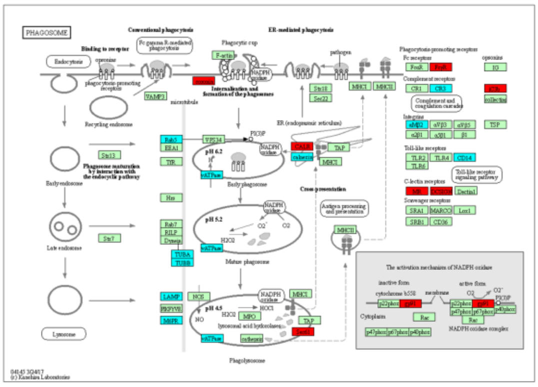

B

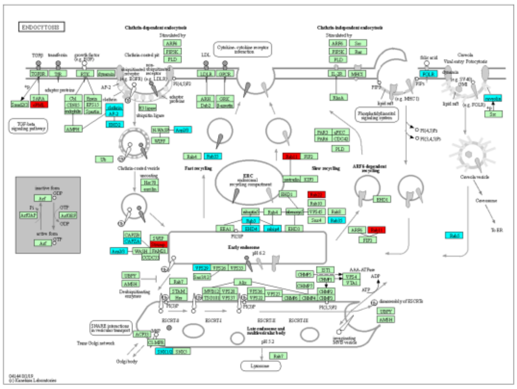

C

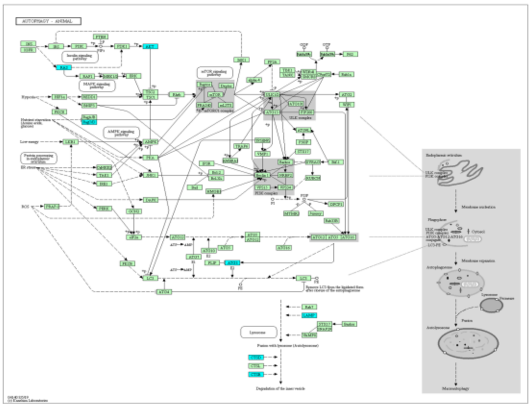

D

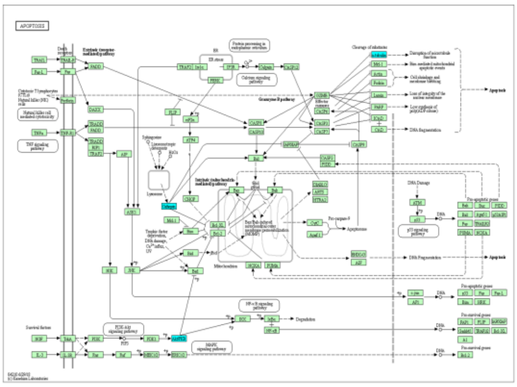

Supplemental Figure 1. Lysosome-associated pathways including phagocytosis (A), endocytosis (B), autophagy (C) and apoptosis (D). Those differentially expressed proteins based on their fold change were coloring with different color: pink ($0 <$ Ratio $\leq 1/3$), yellow ($1/3 <$ Ratio ≤ 0.5), cyan ($2 <$ Ratio ≤ 3) and red (Ratio > 3), light green (background color).

Supplementary figure 2

A

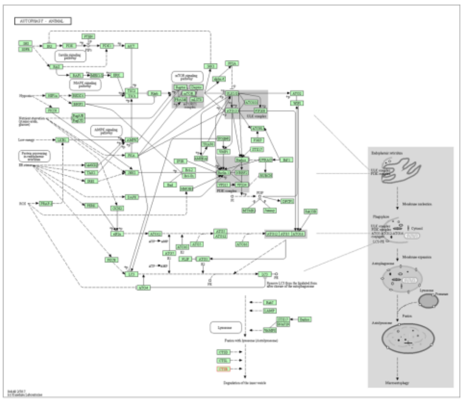

B

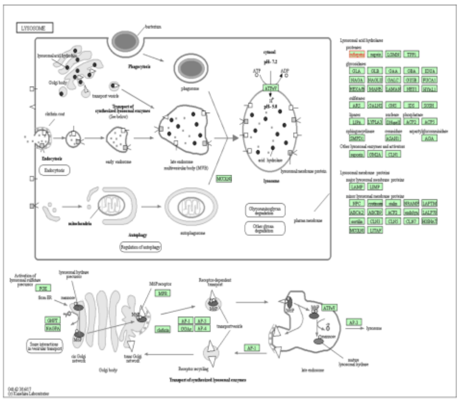

C

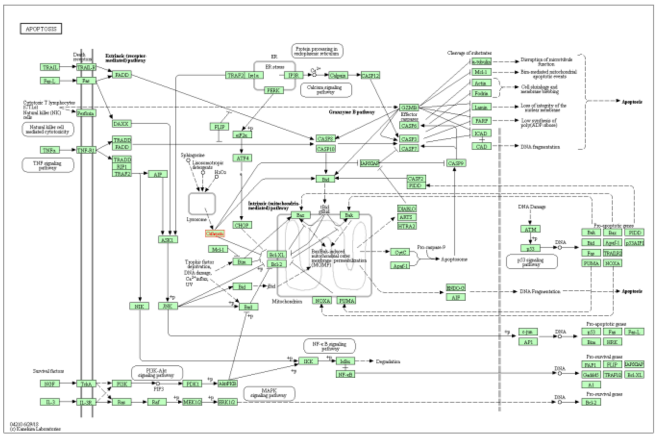

Supplementary Figure 2. *CTSB/CTSD* gene related pathway including autophagy (A), lysosome (B), apoptosis (C)

Supplementary Table 1. Clinical characteristics of patients with deep PU

Gender	Age	Etiology/ Past Disease	Multi- morbidity	Ulceration Duration	Ulceration Position	Ulceration Size(cm^2)	Wound Infection	Sample Use
M	18	Paraplegia	No	5 m	sacrococcygeal	10×18	Yes	proteomics
M	29	Traumatic Brain Injury	No	3m	sacrococcygeal	6×8	Yes	proteomics
M	68	Postoperative Wound Infection	Yes	1m	sacrococcygeal	12×10	Yes	proteomics
F	19	Multiple Trauma	Yes	1m	sacrococcygeal	6×7	Yes	verification
F	29	Syringomyelia	No	12m	ischial tuberosity	5×5	Yes	verification
M	74	Paraplegia	No	3m	sacrococcygeal	11×10	Yes	verification
M	43	Paraplegia	No	6m	ischial tuberosity	11×10	Yes	verification
F	39	Paraplegia	No	3m	sacrococcygeal	8×8	Yes	verification

Supplementary Table 2. List of significant differentially expressed proteins identified by LC–MS/MS analysis between PU muscles and normal muscles

Protein accession	Gene name	Regulated Type	Fold change	P value	MW [kDa]	Score
A0A075B6S6	IGKV2D-30	Up	6.319	0.01557076	13.215	41.6
A0A087X1C5	CYP2D7	Up	14.282	0.00289267	57.488	1.3195
A0A0A0MS15	IGHV3-49	Up	7.018	0.01143165	13.056	5.754
A0A0B4J1V0	IGHV3-15	Up	5.707	0.01977092	12.926	3.4864
A0A0C4DH73	IGKV1-12	Up	4.926	0.00026433	12.645	5.8202
A5YKK6	CNOT1	Up	4.53	0.01965247	266.94	3.0689
A8MU46	SMTNL1	Down	0.303	0.02318126	48.952	18.77
B9A064	IGLL5	Up	3.747	0.03117325	23.063	19.664
O00203	AP3B1	Up	2.794	0.01155906	121.32	6.249
O00299	CLIC1	Up	4.616	0.0007184	26.922	71.266
O00506	STK25	Up	2.272	0.01460727	48.111	2.9096
O00567	NOP56	Up	2.139	0.0199852	66.049	8.9178
O14558	HSPB6	Down	0.432	0.03600829	17.135	15.014
O14656	TOR1A	Up	2.043	0.00476002	37.808	1.7073
O14773	TPP1	Up	2.148	0.00998657	61.247	66.227
O14983	ATP2A1	Down	0.33	0.04856086	110.25	227.33
O15067	PFAS	Up	2.237	0.02640138	144.73	5.5043
O15143	ARPC1B	Up	3.184	0.00892022	40.949	23.703
O15144	ARPC2	Up	2.092	0.02320824	34.333	12.131
O15145	ARPC3	Up	3.614	0.01334229	20.546	7.9736
O15230	LAMA5	Up	2.021	0.00062903	399.73	200.06
O15273	TCAP	Down	0.49	0.00173063	19.051	13.933
O15498	YKT6	Up	2.425	0.01230832	22.417	1.5916
O15511	ARPC5	Up	2.065	0.02764628	16.32	3.7675
O43143	DHX15	Up	3.21	0.00230453	90.932	8.2923
O43286	B4GALT5	Up	2.584	0.02742659	45.118	1.6475
O43390	HNRNPR	Up	2.328	0.00668689	70.942	29.57
O43447	PPIH	Up	2.927	0.00161496	19.208	1.5654
O43708	GSTZ1	Down	0.478	0.00901387	24.212	4.5343
O43795	MYO1B	Up	2.25	0.01175034	131.98	23.571
O43809	NUDT21	Up	2.535	0.02909124	26.227	6.3634
O43866	CD5L	Up	2.625	0.0132238	38.087	25.944
O60264	SMARCA5	Up	3.025	0.00025505	121.9	2.9875
O60269	GPRIN2	Down	0.469	0.00093904	47.45	1.1546
O60488	ACSL4	Up	2.051	0.00131398	79.187	3.5212
O60662	KLHL41	Down	0.483	0.00874555	68.036	101.57
O60674	JAK2	Down	0.309	0.0024996	130.67	1.3937
O75112	LDB3	Down	0.417	0.00656659	77.134	305.47
O75165	DNAJC13	Up	2.62	0.02421005	254.41	5.2659
O75298	RTN2	Down	0.424	0.01017678	59.263	9.3649
O75369	FLNB	Up	2.423	1.0094E-05	278.16	123.58
O75380	NDUFS6	Down	0.318	0.04178747	13.711	12.282
O75643	SNRNP200	Up	2.253	0.00144382	244.5	23.933
O75821	EIF3G	Up	2.031	0.01815299	35.611	6.0193

O75844	ZMPSTE24	Up	2.239	0.02576368	54.812	1.958
O76003	GLRX3	Up	2.434	0.02680272	37.432	6.2247
O94760	DDAH1	Down	0.321	0.01740881	31.121	1.2454
O94973	AP2A2	Up	2.387	0.01891275	103.96	34.928
O95319	CELF2	Up	4.125	0.00549431	54.284	20.731
O95782	AP2A1	Up	2.335	0.01238601	107.54	39.393
P00450	CP	Up	2.827	0.0179134	122.2	303.71
P00488	F13A1	Up	3.476	0.03687199	83.266	130.4
P00734	F2	Up	3.232	0.000125	70.036	193.57
P00747	PLG	Up	2.774	0.01660927	90.568	172.01
P00748	F12	Up	5.856	0.02788744	67.791	10.956
P00751	CFB	Up	3.202	0.00021051	85.532	108.4
P00966	ASS1	Up	2.009	0.03158895	46.53	23.342
P01008	SERPINC1	Up	3.63	0.02775282	52.602	36.657
P01009	SERPINA1	Up	4.137	0.01327771	46.736	163.32
P01011	SERPINA3	Up	3.597	0.02146316	47.65	99.596
P01023	A2M	Up	3.069	0.03102428	163.29	323.31
P01024	C3	Up	4.159	0.00790021	187.15	323.31
P01031	C5	Up	2.996	0.00906237	188.3	38.682
P01042	KNG1	Up	2.39	0.0128318	71.957	124.47
P01591	JCHAIN	Up	2.189	0.00320425	18.098	11.104
P01602	IGKV1-5	Up	3.126	0.00365276	12.781	6.9229
P01619	IGKV3-20	Up	3.655	0.0006059	12.557	68.917
P01624	IGKV3-15	Up	2.955	0.01831282	12.496	41.098
P01700	IGLV1-47	Up	2.653	0.04179168	12.283	14.865
P01701	IGLV1-51	Up	2.028	0.02778454	12.249	2.407
P01772	IGHV3-33	Up	2.214	0.02922016	13.074	8.1883
P01780	IGHV3-7	Up	5.297	0.0382216	12.943	4.1985
P01825	IGHV4-59	Up	2.872	0.00772992	12.936	80.426
P01834	IGKC	Up	5.606	0.04852105	11.609	301
P01857	IGHG1	Up	3.875	0.04443497	36.105	252.78
P01859	IGHG2	Up	4.824	0.00106761	35.9	164.85
P01871	IGHM	Up	5.018	0.03373044	49.306	170.94
P02671	FGA	Up	3.667	0.01452218	94.972	323.31
P02675	FGB	Up	4.548	0.01518341	55.928	323.31
P02679	FGG	Up	4.133	0.01462458	51.511	260.72
P02743	APCS	Up	2.789	0.02948304	25.387	64.083
P02745	C1QA	Up	2.661	0.01783732	26.016	12.895
P02747	C1QC	Up	2.182	0.00460279	25.773	15.195
P02748	C9	Up	3.229	0.00149456	63.173	40.467
P02749	APOH	Up	3.08	3.0948E-05	38.298	100.61
P02760	AMBP	Up	4.753	0.0046666	38.999	50.523
P02774	GC	Up	3.184	0.01089086	52.963	141.99
P02787	TF	Up	3.461	0.01453493	77.063	323.31
P02790	HPX	Up	2.824	0.00019535	51.676	205.99
P03952	KLKB1	Up	3.198	0.01553409	71.369	9.8646
P04003	C4BPA	Up	3.211	0.00401935	67.033	97.554
P04004	VTN	Up	2.846	0.03072619	54.305	95.79

P04075	ALDOA	Down	0.446	0.02435941	39.42	323.31
P04083	ANXA1	Up	3.716	0.02432381	38.714	219.21
P04196	HRG	Up	2.914	0.04727062	59.578	14.424
P04216	THY1	Up	4.318	0.04489696	17.935	52.459
P04217	A1BG	Up	5.934	0.02315521	54.253	77.632
P04275	VWF	Up	2.067	0.01686671	309.26	106.55
P04433	IGKV3-11	Up	2.555	0.00077185	12.575	18.065
P04839	CYBB	Up	8.208	0.02207822	65.335	10.936
P04843	RPN1	Up	2.467	0.0463595	68.569	73.114
P04844	RPN2	Up	3.163	0.00339269	69.283	72.545
P04899	GNAI2	Up	3.102	0.04143888	40.45	119.97
P05023	ATP1A1	Up	2.028	0.01171534	112.89	69.446
P05107	ITGB2	Up	2.768	0.01056586	84.781	18.159
P05109	S100A8	Up	3.389	0.02202651	10.834	23.444
P05141	SLC25A5	Up	3.269	0.02915325	32.852	10.729
P05155	SERPING1	Up	3.625	0.02581296	55.154	141.36
P05386	RPLP1	Up	2.528	0.00096816	11.514	8.3638
P05387	RPLP2	Up	2.583	0.0051363	11.665	26.402
P05388	RPLP0	Up	2.044	0.04008007	34.273	50.187
P05546	SERPIND1	Up	2.866	0.00614184	57.07	19.031
P05976	MYL1	Down	0.299	0.02435905	21.145	254.17
P06280	GLA	Up	4.42	0.01276167	48.766	10.82
P06312	IGKV4-1	Up	5.445	0.02292408	13.38	16.988
P06681	C2	Up	2.097	0.00311878	83.267	25.912
P06702	S100A9	Up	4.63	0.00735743	13.242	64.576
P06732	CKM	Down	0.409	0.01362858	43.101	323.31
P06737	PYGL	Up	2.136	0.01669006	97.147	10.679
P06748	NPM1	Up	2.755	0.04094293	32.575	63.259
P06753	TPM3	Down	0.333	0.01929812	32.95	18.256
P07225	PROS1	Up	2.783	0.00148898	75.122	1.2417
P07237	P4HB	Up	2.882	0.03581221	57.116	59.093
P07339	CTSD	Up	2.67	0.03268552	44.552	63.466
P07355	ANXA2	Up	3.066	0.04264468	38.604	223.35
P07357	C8A	Up	3.056	0.00186823	65.163	42.754
P07360	C8G	Up	2.511	0.01074131	22.277	38.681
P07437	TUBB	Up	2.81	0.00700829	49.67	52.396
P07451	CA3	Down	0.437	0.03363044	29.557	204.51
P07602	PSAP	Up	3.15	0.03396598	58.112	8.3509
P07741	APRT	Up	2.532	0.0040926	19.608	16.498
P07858	CTSB	Up	2.611	0.01333507	37.821	120.01
P07942	LAMB1	Up	2.112	0.00980016	198.04	96.072
P07948	LYN	Up	2.014	0.00179715	58.573	1.9022
P08134	RHOC	Up	3.681	0.04901472	22.006	11.019
P08195	SLC3A2	Up	4.313	0.00010921	67.993	13.622
P08237	PFKM	Down	0.389	0.02484258	85.182	323.31
P08240	SRPRA	Up	2.514	0.03158068	69.81	5.9682
P08519	LPA	Up	4.731	0.01002446	501.31	8.3289
P08571	CD14	Up	2.285	0.01456334	40.076	28.937

P08575	PTPRC	Up	2.028	0.00653895	147.25	14.795
P08603	CFH	Up	2.484	3.033E-05	139.09	239.03
P08637	FCGR3A	Up	3.134	0.0366777	29.089	6.3553
P08670	VIM	Up	3.678	0.02068515	53.651	208.65
P08697	SERPINF2	Up	3.316	0.00315904	54.565	13.032
P08865	RPSA	Up	2.733	0.03812248	32.854	53.47
P08962	CD63	Up	4.461	0.02464256	25.636	1.7566
P09104	ENO2	Down	0.37	0.01680859	47.268	3.1164
P09211	GSTP1	Up	2.043	0.0392196	23.356	88.588
P09525	ANXA4	Up	2.449	0.01636963	35.882	131.82
P09619	PDGFRB	Up	3.135	0.02480754	123.97	6.7486
P09651	HNRNPA1	Up	2.406	0.02988509	38.746	163.57
P09668	CTSH	Up	3.002	0.01941352	37.393	10.588
P09871	C1S	Up	4.451	0.00044088	76.684	40.521
P0C0L4	C4A	Up	11.94	0.04419296	192.78	30.119
P0CG06	IGLC3	Up	4.272	0.03350989	11.237	135.23
P10301	RRAS	Up	2.988	0.00379607	23.48	31.421
P10643	C7	Up	3.223	0.02066373	93.517	27.342
P10909	CLU	Up	3.704	0.02083914	52.494	52.626
P11021	HSPA5	Up	2.295	0.03120967	72.332	241.55
P11169	SLC2A3	Up	7.717	0.01960832	53.924	1.9545
P11215	ITGAM	Up	2.664	0.03360994	127.18	12.438
P11217	PYGM	Down	0.228	0.02132219	97.091	323.31
P11279	LAMP1	Up	2.43	0.01011411	44.882	15.407
P11413	G6PD	Up	2.617	0.00188469	59.256	27.576
P11498	PC	Up	2.148	0.00366516	129.63	3.7943
P12109	COL6A1	Up	2.369	0.02083513	108.53	323.31
P12111	COL6A3	Up	2.958	0.02915641	343.67	323.31
P12235	SLC25A4	Down	0.232	0.00717447	33.064	193.38
P12236	SLC25A6	Up	2.444	0.02221065	32.866	22.778
P12829	MYL4	Up	4.349	0.04782543	21.564	3.4584
P12956	XRCC6	Up	2.972	0.02623624	69.842	44.074
P13010	XRCC5	Up	2.065	0.01258978	82.704	46.282
P13284	IFI30	Up	2.854	0.02466637	27.963	16.248
P13473	LAMP2	Up	3.8	0.01671864	44.96	1.5096
P13611	VCAN	Up	3.962	0.0402073	372.82	60.004
P13667	PDIA4	Up	2.802	0.01332715	72.932	57.751
P13796	LCP1	Up	3.085	0.00724426	70.288	92.359
P13929	ENO3	Down	0.295	0.02750557	46.986	232.73
P14174	MIF	Up	2.18	0.03775571	12.476	6.0667
P14207	FOLR2	Up	2.8	0.01581203	29.279	5.4781
P14543	NID1	Up	2.359	0.00215393	136.38	180.94
P14621	ACYP2	Down	0.445	0.01277705	11.139	9.4935
P14625	HSP90B1	Up	3.334	0.03041711	92.468	139.93
P14649	MYL6B	Down	0.392	0.02032113	22.764	76.132
P14780	MMP9	Up	2.129	0.03514507	78.457	12.923
P14868	DARS	Up	2.062	0.00835801	57.136	25.734
P15144	ANPEP	Up	2.639	0.00145992	109.54	34.078

P15169	CPN1	Up	2.685	0.00956278	52.286	1.4063
P15259	PGAM2	Down	0.309	0.0334598	28.766	19.23
P15531	NME1	Up	2.231	0.00035766	17.149	2.0142
P16070	CD44	Up	4.792	0.00210676	81.537	11.326
P16435	POR	Up	2.188	0.01953551	76.689	7.7918
P17174	GOT1	Down	0.445	0.00426225	46.247	143.05
P17813	ENG	Up	2.073	0.00015637	70.577	1.2136
P18564	ITGB6	Up	2.226	0.01521547	85.935	2.1272
P18669	PGAM1	Up	2.143	0.04934348	28.804	76.394
P19105	MYL12A	Up	3.014	0.02022879	19.794	3.937
P19338	NCL	Up	2.389	0.0476355	76.613	17.544
P19823	ITIH2	Up	2.964	0.01876928	106.46	88.607
P19827	ITIH1	Up	2.399	0.04237117	101.39	92.618
P19971	TYMP	Up	2.778	0.00049043	49.955	60.507
P20020	ATP2B1	Up	2.419	0.01410153	138.75	6.6696
P20645	M6PR	Up	2.749	0.03134957	30.993	4.0301
P20851	C4BPB	Up	5.342	0.00778939	28.357	9.0227
P20929	NEB	Down	0.298	2.9595E-05	772.91	323.31
P21281	ATP6V1B2	Up	2.558	0.00950137	56.5	30.541
P21810	BGN	Up	3.274	0.02607764	41.654	103.75
P21980	TGM2	Up	3.588	0.0117911	77.328	94.679
P22307	SCP2	Up	2.858	0.01092433	58.993	11.216
P22352	GPX3	Up	2.068	0.00933725	25.552	17.723
P22392	NME2	Up	2.256	0.01433923	17.298	36.75
P22626	HNRNPA2B1	Up	2.012	0.00326077	37.429	67.477
P22897	MRC1	Up	3.155	0.00365321	166.01	19.845
P23109	AMPD1	Down	0.398	0.00081462	90.218	127.35
P23193	TCEA1	Up	3.362	0.01086208	33.969	2.3019
P23526	AHCY	Up	2.092	0.00039538	47.716	11.184
P23528	CFL1	Up	2.573	0.03798188	18.502	47.498
P24557	TBXAS1	Up	2.924	0.0073369	60.518	9.8364
P25311	AZGP1	Up	2.38	0.04014418	34.258	67.046
P25398	RPS12	Up	2.142	0.028905	14.515	10.381
P26022	PTX3	Up	6.001	0.01535376	41.975	7.2406
P26038	MSN	Up	3.431	0.03474376	67.819	89.828
P26368	U2AF2	Up	3.012	0.02187227	53.5	12.057
P26447	S100A4	Up	2.354	0.03806946	11.728	6.6624
P26583	HMGB2	Up	16.347	0.04689844	24.033	-2
P27105	STOM	Up	2.077	0.02107373	31.73	33.162
P27169	PON1	Up	5.696	0.02572684	39.731	35.191
P27348	YWHAQ	Up	10.228	0.02228616	27.764	8.5002
P27695	APEX1	Up	2.69	0.00913856	35.554	52.672
P27708	CAD	Up	4.005	0.01924685	242.98	3.8504
P27797	CALR	Up	3.098	0.00429214	48.141	25.974
P27824	CANX	Up	2.226	0.0289769	67.567	37.264
P27918	CFP	Up	2.475	0.0004225	51.276	4.6093
P28161	GSTM2	Down	0.465	0.02303078	25.744	56.997
P28799	GRN	Up	2.551	0.0024784	63.544	2.3957

P29350	PTPN6	Up	4.066	0.00497445	67.56	2.3598
P29401	TKT	Up	3.731	0.00581674	67.877	101.23
P29590	PML	Up	3.329	0.04207967	97.55	15.607
P29622	SERPINA4	Up	2.355	0.02357951	48.541	6.4172
P30101	PDIA3	Up	3.545	0.03124512	56.782	94.4
P30520	ADSS	Up	3.2	0.0134624	50.097	7.7392
P30711	GSTT1	Down	0.281	0.00472421	27.335	-2
P31146	CORO1A	Up	4.327	0.0178683	51.026	19.004
P31415	CASQ1	Down	0.442	0.01067849	45.16	47.461
P31749	AKT1	Up	2.112	0.02232343	55.686	1.7187
P31943	HNRNPH1	Up	2.306	0.01397344	49.229	80.279
P31946	YWHAB	Up	2.07	0.03017056	28.082	26.673
P31949	S100A11	Up	5.475	0.00590415	11.74	22.313
P35573	AGL	Down	0.461	0.00304184	174.76	270.44
P35579	MYH9	Up	2.778	0.029703	226.53	274.59
P35606	COPB2	Up	2.672	0.04495142	102.49	45.513
P35609	ACTN2	Down	0.29	0.00286796	103.85	323.31
P35637	FUS	Up	2.187	0.04918491	53.425	3.1708
P35858	IGFALS	Up	3.258	0.02646677	66.034	17.185
P36871	PGM1	Down	0.482	0.00633811	61.448	108.82
P37837	TALDO1	Up	2.616	0.00096668	37.54	28.874
P38919	EIF4A3	Up	2.199	0.01367337	46.871	6.3574
P39060	COL18A1	Up	2.604	0.00134833	178.19	93.436
P40261	NNMT	Up	4.412	0.04883256	29.574	32.457
P41240	CSK	Up	4.057	0.00366976	50.704	2.4364
P41252	IARS	Up	2.15	0.00584901	144.5	25.829
P42167	TMPO	Up	2.395	0.02565315	50.67	1.9188
P42224	STAT1	Up	2.475	0.00103246	87.334	31.593
P42285	SKIV2L2	Up	2.854	0.00834193	117.8	1.9671
P46940	IQGAP1	Up	2.197	0.03748071	189.25	85.767
P46977	STT3A	Up	2.552	0.03709081	80.529	4.083
P48444	ARCN1	Up	2.538	0.04621244	57.21	32.399
P49354	FNTA	Up	2.207	0.0021978	44.408	1.8559
P49591	SARS	Up	2.798	0.00760036	58.777	13.065
P49754	VPS41	Up	3.463	0.03683383	98.565	11.396
P49755	TMED10	Up	2.762	0.00701116	24.976	19.197
P49821	NDUFV1	Down	0.482	0.03926111	50.817	116.08
P50914	RPL14	Up	2.415	0.04217759	23.432	12.439
P50993	ATP1A2	Down	0.409	0.01600252	112.26	18.238
P51148	RAB5C	Up	2.917	0.00229229	23.482	5.3761
P51649	ALDH5A1	Down	0.279	0.0052713	57.214	18.888
P51659	HSD17B4	Up	2.379	0.00192756	79.685	10.306
P51991	HNRNPA3	Up	2.87	0.01361323	39.594	47.583
P52179	MYOM1	Down	0.33	0.00045257	187.62	323.31
P52209	PGD	Up	2.757	0.00896441	53.139	51.682
P52272	HNRNPM	Up	2.057	0.02475113	77.515	61.85
P52566	ARHGDIB	Up	3.942	0.00651063	22.988	18.048
P52594	AGFG1	Up	2.225	0.00035244	58.259	3.6458

P52597	HNRNPF	Up	2.886	0.00476227	45.671	8.5404
P52790	HK3	Up	2.271	0.00516218	99.024	4.1875
P52907	CAPZA1	Up	2.219	0.01405063	32.922	49.199
P53396	ACLY	Up	2.889	0.01835082	120.84	29.559
P53634	CTSC	Up	2.045	0.04234072	51.853	20.02
P54136	RARS	Up	2.38	0.02671888	75.378	34.009
P54296	MYOM2	Down	0.315	0.01232401	164.89	323.31
P54577	YARS	Up	2.112	0.00631789	59.143	4.8496
P54709	ATP1B3	Up	5.641	0.01252745	31.512	7.8842
P55822	SH3BGR	Down	0.271	0.00400688	26.085	8.696
P56537	EIF6	Up	2.075	0.02411631	26.599	11.539
P59998	ARPC4	Up	2.706	0.01364375	19.667	9.9181
P60468	SEC61B	Up	4.731	0.0265607	9.9743	11.223
P60903	S100A10	Up	2.668	0.01269421	11.203	19.112
P60983	GMFB	Up	2.332	0.00102822	16.713	3.1511
P61158	ACTR3	Up	3.068	0.0177495	47.371	46.919
P61160	ACTR2	Up	2.008	0.00313242	44.76	26.308
P61224	RAP1B	Up	2.304	0.00998332	20.825	21.746
P61225	RAP2B	Up	4.38	0.02141084	20.504	14.73
P61626	LYZ	Up	3.35	0.01934377	16.537	47.755
P61769	B2M	Up	2.895	0.00038381	13.714	4.0265
P61916	NPC2	Up	2.257	0.00345964	16.57	11.167
P61978	HNRNPK	Up	3.784	0.00629446	50.976	27.915
P62244	RPS15A	Up	2.159	0.0268642	14.839	12.835
P62310	LSM3	Up	2.172	0.01578734	11.845	5.2019
P62873	GNB1	Up	2.19	0.01260994	37.377	90.202
P62913	RPL11	Up	2.276	0.04159146	20.252	8.2994
P62937	PPIA	Up	2.623	0.00978892	18.012	20.134
P63104	YWHAZ	Up	3.358	0.01929959	27.745	40.02
P63162	SNRPN	Up	2.577	0.00347916	24.614	1.729
P67870	CSNK2B	Up	2.297	0.04862737	24.942	4.5454
P68133	ACTA1	Down	0.406	0.00010016	42.051	323.31
P78344	EIF4G2	Up	2.169	0.03896707	102.36	6.9763
P78527	PRKDC	Up	2.059	0.0085062	469.08	57.327
P80748	IGLV3-21	Up	4.829	0.01681199	12.446	57.894
P83111	LACTB	Down	0.433	0.00132136	60.693	9.9277
P98171	ARHGAP4	Up	3.679	0.00532921	105.02	11.904
Q00535	CDK5	Up	2.11	0.00151235	33.304	1.5588
Q00610	CLTC	Up	2.679	0.02188644	191.61	292.98
Q00872	MYBPC1	Down	0.233	0.00158739	128.29	323.31
Q01469	FABP5	Up	3.117	0.03403024	15.164	116.18
Q01518	CAP1	Up	2.369	0.01397683	51.901	30.498
Q02809	PLOD1	Up	4.323	0.03452589	83.549	10.568
Q03135	CAV1	Up	2.681	0.04374455	20.471	30.205
Q03591	CFHR1	Up	2.178	0.04296223	37.65	11.178
Q06033	ITIH3	Up	2.244	0.02476709	99.848	7.0678
Q06278	AOX1	Up	2.264	0.02553131	147.92	3.4229
Q06323	PSME1	Up	2.337	0.00869987	28.723	12.98

Q07954	LRP1	Up	2.554	0.0382928	504.6	118.99
Q08380	LGALS3BP	Up	2.793	0.01610172	65.33	21.095
Q08945	SSRP1	Up	3.642	0.04284961	81.074	8.8455
Q10471	GALNT2	Up	2.765	0.0079369	64.732	5.8882
Q12768	KIAA0196	Up	3.289	0.00224096	134.28	7.7053
Q12797	ASPH	Up	3.461	0.02402027	85.862	38.805
Q12905	ILF2	Up	2.057	0.00138593	43.062	21.98
Q12965	MYO1E	Up	2.328	0.0059581	127.06	10.669
Q13151	HNRNPA0	Up	2.995	0.02247771	30.84	8.6445
Q13247	SRSF6	Up	2.673	0.0359275	39.586	4.1906
Q13263	TRIM28	Up	2.956	0.010881	88.549	28.43
Q13283	G3BP1	Up	2.107	0.02520329	52.164	19.34
Q13492	PICALM	Up	5.514	0.00562587	70.754	15.09
Q13508	ART3	Down	0.473	0.00123211	43.923	8.0292
Q13509	TUBB3	Up	2.387	0.03451978	50.432	34.676
Q13510	ASAH1	Up	2.631	0.03308414	44.659	18.029
Q13555	CAMK2G	Down	0.488	0.00178003	62.608	2.7706
Q13595	TRA2A	Up	2.234	0.02823269	32.688	3.3123
Q13596	SNX1	Up	2.505	0.01269768	59.069	15.73
Q13636	RAB31	Up	4.091	0.0483275	21.569	15.828
Q13642	FHL1	Down	0.244	7.5328E-05	36.263	231.44
Q13643	FHL3	Down	0.401	0.02362688	31.192	63.445
Q14019	COTL1	Up	2.152	0.01944661	15.945	4.3925
Q14103	HNRNPD	Up	2.243	0.02057168	38.434	18.68
Q14108	SCARB2	Up	2.551	0.00047073	54.29	14.686
Q14166	TTLL12	Up	2.398	0.01744989	74.403	5.8654
Q14247	CTTN	Up	2.08	0.00522379	61.585	10.875
Q14697	GANAB	Up	2.77	0.02680641	106.87	46.154
Q14764	MVP	Up	2.375	0.00341317	99.326	94.576
Q15020	SART3	Up	2.007	0.03121316	109.93	4.3787
Q15052	ARHGEF6	Up	3.023	0.03335012	87.498	2.6729
Q15084	PDIA6	Up	3.225	0.00147421	48.121	177.93
Q15102	PAFAH1B3	Up	2.219	0.00430536	25.734	5.5408
Q15111	PLCL1	Down	0.264	0.00526096	122.73	1.4525
Q15172	PPP2R5A	Down	0.368	0.00530243	56.193	4.1238
Q15185	PTGES3	Up	2.272	0.0241639	18.697	30.136
Q15233	NONO	Up	2.113	0.04097401	54.231	46.348
Q15286	RAB35	Up	2.202	0.03187788	23.025	4.989
Q15293	RCN1	Up	2.365	0.01299043	38.89	3.0872
Q15424	SAFB	Up	2.413	0.01604559	102.64	4.7499
Q15907	RAB11B	Up	3.247	0.00497162	24.488	10.88
Q16181	SEPT7	Up	5.266	0.04391598	50.679	26.201
Q16363	LAMA4	Up	2.707	0.01458848	202.52	71.251
Q16401	PSMD5	Up	2.554	0.01408255	56.195	28.849
Q16610	ECM1	Up	2.238	0.00354955	60.673	26.793
Q16718	NDUFA5	Down	0.364	0.04943714	13.459	15.486
Q16795	NDUFA9	Down	0.459	0.04950808	42.509	62.018
Q16821	PPP1R3A	Down	0.36	0.03234313	125.77	3.4082

Q16853	AOC3	Up	2.158	0.04946302	84.621	83.404
Q1KMD3	HNRNPUL2	Up	2.873	0.0360712	85.104	23.783
Q27J81	INF2	Up	4.567	0.02571005	135.62	1.1153
Q53GG5	PDLIM3	Down	0.403	0.00155992	39.232	264.89
Q53T59	HS1BP3	Up	2.18	0.04064565	42.78	12.08
Q5BN46	C9orf116	Down	0.156	0.03189476	15.26	1.2531
Q5HYK3	COQ5	Down	0.46	0.04502492	37.14	23.058
Q5SYB0	FRMPD1	Up	2.549	0.03485892	173.43	1.2585
Q5T447	HECTD3	Down	0.213	0.01497266	97.112	2.2658
Q5TDH0	DDI2	Up	3.695	0.03724163	44.522	5.3519
Q5TFQ8	SIRPB1	Up	2.181	0.00663661	43.359	8.8049
Q5VTE0	EEF1A1P5	Up	2.167	0.0040778	50.184	24.203
Q5VTT5	MYOM3	Down	0.321	0.01281365	162.19	323.31
Q687X5	STEAP4	Up	2.124	0.00729903	51.981	36.877
Q6NUK1	SLC25A24	Up	2.15	0.00070018	53.354	14.07
Q6P2Q9	PRPF8	Up	3.089	0.01490269	273.6	12.778
Q6PCB0	VWA1	Up	2.504	0.02823573	46.804	23.537
Q6QEF8	CORO6	Down	0.486	0.03055554	52.761	17.8
Q6UVK1	CSPG4	Up	2.226	0.00325428	250.53	14.175
Q6VY07	PACS1	Up	2.581	0.00247664	104.9	2.9513
Q6ZSR9	---	Up	2.008	0.00913008	37.976	4.8349
Q86SZ2	TRAPPC6B	Down	0.178	0.00059978	17.983	1.3749
Q86TD4	SRL	Down	0.412	0.00143334	100.79	129
Q86UD0	SAPCD2	Down	0.176	8.5502E-05	42.636	1.6567
Q86UP2	KTN1	Up	2.099	0.01934084	156.27	5.6957
Q86UX7	FERMT3	Up	2.131	0.00164784	75.952	28.01
Q86VB7	CD163	Up	3.923	0.00936318	125.45	68.576
Q86Y39	NDUFA11	Down	0.413	0.00112147	14.852	18.485
Q8IVL6	P3H3	Up	4.115	0.03487475	81.836	2.3186
Q8IVN3	MUSTN1	Down	0.36	0.04961759	8.911	49.05
Q8N163	CCAR2	Up	3.469	0.04374516	102.9	40.501
Q8N3V7	SYNPO	Down	0.444	0.00728673	99.462	43.795
Q8N4P2	TTC30B	Down	0.369	0.00010625	76.098	1.2636
Q8N5K1	CISD2	Down	0.229	0.00111117	15.278	6.9099
Q8N944	AMER3	Up	3.606	0.04929531	90.444	1.3182
Q8NB12	SMYD1	Down	0.447	0.00328943	56.616	70.984
Q8NBQ5	HSD17B11	Up	2.542	0.00644948	32.935	7.6482
Q8TC12	RDH11	Up	2.328	0.00942013	35.386	2.7972
Q8TCA0	LRRC20	Down	0.429	0.00335866	20.509	17.72
Q8TDC0	MYOZ3	Down	0.383	0.03310439	27.157	31.14
Q8TDZ2	MICAL1	Up	3.592	0.03248121	117.87	1.825
Q8TEX9	IPO4	Up	2.182	0.02212696	118.71	4.0075
Q8WUW1	BRK1	Up	3.578	0.00218347	8.7448	3.4612
Q8WZ42	TTN	Down	0.341	0.00046512	3816	323.31
Q92626	PXDN	Up	4.009	0.00764427	165.27	21.251
Q92736	RYR2	Up	17.648	0.01224355	564.56	2.2837
Q92882	OSTF1	Up	2.656	0.01899168	23.787	2.875
Q92930	RAB8B	Up	4.586	0.00296158	23.584	25.359

Q93100	PHKB	Down	0.348	0.04528054	124.88	9.8097
Q969V5	MUL1	Down	0.28	0.00055545	39.8	1.1456
Q96AE4	FUBP1	Up	2.015	0.01304535	67.56	9.2879
Q96C19	EFHD2	Up	3.836	0.00085728	26.697	19.213
Q96CW1	AP2M1	Up	2.477	0.00331439	49.654	10.879
Q96DG6	CMBL	Down	0.417	0.00370372	28.048	36.036
Q96FQ6	S100A16	Up	2.684	0.01481646	11.801	2.8373
Q96HC4	PDLIM5	Down	0.36	9.8812E-06	63.944	189.97
Q96HE7	ERO1A	Up	3.607	0.00808149	54.392	8.1471
Q96I99	SUCLG2	Up	2.096	2.9278E-05	46.51	12.644
Q96IJ6	GMPPA	Up	2.306	0.02758183	46.291	10.583
Q96IY4	CPB2	Up	2.198	0.00676778	48.424	7.6713
Q96K21	ZFYVE19	Down	0.175	0.00010373	51.546	1.258
Q96KP4	CNDP2	Up	2.856	0.04210513	52.878	30.989
Q96MF6	COQ10A	Down	0.343	0.00831126	27.686	4.8148
Q96PD5	PGLYRP2	Up	6.901	0.03789978	62.216	20.972
Q96T51	RUFY1	Up	2.757	0.01498946	79.817	10.307
Q96TA1	FAM129B	Up	2.587	0.02576664	84.137	13.568
Q99538	LGMN	Up	4.891	0.00315002	49.411	48.463
Q99729	HNRNPAB	Up	2.363	0.03187966	36.224	7.3315
Q99829	CPNE1	Up	3.523	0.02948769	59.058	11.389
Q9BPW8	NIPSNAP1	Up	5.401	0.01569947	33.31	12.721
Q9BQE3	TUBA1C	Up	2.832	0.0064226	49.895	8.4808
Q9BRA2	TXNDC17	Up	2.214	0.00179483	13.941	27.007
Q9BS26	ERP44	Up	2.059	0.01702788	46.971	18.803
Q9BSJ8	ESYT1	Up	2.201	0.00128316	122.85	39.881
Q9BUF5	TUBB6	Up	2.346	0.00256664	49.857	61.971
Q9BUJ2	HNRNPUL1	Up	2.233	0.00294113	95.737	2.7509
Q9BVA1	TUBB2B	Up	2.185	0.03411902	49.953	60.544
Q9BVK6	TMED9	Up	2.09	0.01606076	27.277	10.372
Q9BWD1	ACAT2	Up	4.381	0.0134543	41.35	71.797
Q9BWM7	SFXN3	Up	2.114	0.02026409	35.978	13.525
Q9BYT3	STK33	Down	0.365	0.00676962	57.83	1.6676
Q9C0C2	TNKS1BP1	Up	2.436	0.00337703	181.79	15.476
Q9GZV1	ANKRD2	Down	0.32	0.02092231	39.859	120.1
Q9H1R3	MYLK2	Down	0.495	0.0042401	64.684	15.387
Q9H223	EHD4	Up	2.531	0.00371228	61.174	21.866
Q9H299	SH3BGRL3	Up	2.208	0.00336256	10.438	37.251
Q9H2M9	RAB3GAP2	Up	2.799	1.4306E-05	155.98	1.9093
Q9H2U2	PPA2	Up	2.09	0.00857797	37.92	2.2806
Q9H3N1	TMX1	Up	2.261	0.0188271	31.791	3.629
Q9H4G4	GLIPR2	Up	2.19	0.00633173	17.218	12.148
Q9H7C9	AAMDC	Down	0.327	0.01800191	13.332	9.9436
Q9HB90	RRAGC	Up	2.068	0.03568771	44.223	1.3595
Q9HDC9	APMAP	Up	2.878	0.00826641	46.48	9.3277
Q9NNX6	CD209	Up	4.238	0.04200031	45.774	4.0101
Q9NP98	MYOZ1	Down	0.264	0.00156192	31.744	195.2
Q9NRP0	OSTC	Up	2.546	0.02071854	16.829	4.0032

Q9NRV9	HEBP1	Up	2.349	0.00048001	21.097	4.3492
Q9NRW1	RAB6B	Up	2.141	0.00248842	23.461	6.3692
Q9NT62	ATG3	Up	2.464	0.00107399	35.864	6.0453
Q9NTX5	ECHDC1	Up	2.508	0.02353332	33.698	4.247
Q9NUQ9	FAM49B	Up	2.767	0.00362177	36.748	21.934
Q9NY15	STAB1	Up	2.747	0.03571578	275.48	62.231
Q9NYL9	TMOD3	Up	2.14	0.00895434	39.594	1.5115
Q9NYU2	UGGT1	Up	2.818	0.00533004	177.19	18.822
Q9NZ08	ERAP1	Up	2.092	0.0264843	107.23	33.394
Q9NZD4	AHSP	Down	0.32	0.03148745	11.84	3.1787
Q9NZJ7	MTCH1	Up	2.207	0.01278967	41.544	2.9768
Q9NZJ9	NUDT4	Up	2.729	2.0255E-05	20.306	1.8227
Q9NZN4	EHD2	Up	2.065	0.03909639	61.161	61.783
Q9NZQ9	TMOD4	Down	0.307	0.00910152	39.335	10.821
Q9NZU5	LMCD1	Down	0.399	0.02338421	40.832	79.532
Q9P0V3	SH3BP4	Up	19.282	0.04014294	107.49	1.1757
Q9P1F3	ABRACL	Up	7.213	0.01314707	9.0564	2.9801
Q9UBF9	MYOT	Down	0.492	0.00597209	55.395	207.22
Q9UBI1	COMMD3	Up	2.216	0.01233537	22.151	17.658
Q9UBQ0	VPS29	Up	2.577	0.0006576	20.505	6.6695
Q9UBR2	CTSZ	Up	5.011	0.02318923	33.868	9.9198
Q9UBX5	FBLN5	Up	2.633	0.04704674	50.18	7.7013
Q9UEY8	ADD3	Up	2.053	0.02881221	79.154	7.7607
Q9UHD8	SEPT9	Up	2.134	0.03293295	65.401	20.343
Q9UHX1	PUF60	Up	2.133	0.00081495	59.875	1.1914
Q9UJ70	NAGK	Up	2.255	0.03494991	37.375	20.99
Q9UJU6	DBNL	Up	2.454	0.02704495	48.207	24.301
Q9UKX2	MYH2	Down	0.233	0.04094015	223.04	323.31
Q9UKX3	MYH13	Down	0.257	0.0002883	223.6	2.677
Q9UMS4	PRPF19	Up	2.159	0.02166477	55.18	3.4625
Q9UQM7	CAMK2A	Down	0.485	0.00713577	54.087	8.1706
Q9Y224	C14orf166	Up	2.17	0.00368575	28.068	2.9526
Q9Y235	APOBEC2	Down	0.367	0.02889202	25.703	72.26
Q9Y281	CFL2	Down	0.263	2.1414E-05	18.736	19.084
Q9Y2D4	EXOC6B	Down	0.368	0.02900089	94.2	4.0443
Q9Y2T2	AP3M1	Up	2.445	0.00981764	46.939	2.3881
Q9Y3L3	SH3BP1	Up	2.87	0.00321421	75.712	3.2918
Q9Y3Z3	SAMHD1	Up	2.395	0.02392255	72.2	29.751
Q9Y490	TLN1	Up	2.566	0.0383044	269.76	320.83
Q9Y4W6	AFG3L2	Down	0.477	0.01032568	88.583	8.5979
Q9Y5F9	PCDHGB6	Down	0.373	0.00024312	101.04	1.216
Q9Y625	GPC6	Up	2.327	0.04533402	62.735	5.0174
Q9Y6C2	EMILIN1	Up	2.782	0.02451884	106.67	116.22
Q9Y6Y8	SEC23IP	Up	2.093	0.01468119	111.08	1.6122
